# A systematic evaluation of the Olympus AU5061 as an effective replacement for the SMAC II analyser

**DOI:** 10.1155/S1463924688000161

**Published:** 1988

**Authors:** Philip B. Hodgin, William F. Moore, Harvey L. Kincaid

**Affiliations:** SKBL-Tampa SmithKline BioScience Laboratories Ltd P.O. Box 22707 Tampa Florida 33630 USA


Journal of Automatic Chemistry, Vol. 10, No. 2 (April–June 1988), pp. 79–87

### A systematic evaluation of the Olympus AU5061 as an effective replacement for the SMAC II analyser

**Philip B. Hodgin, Jr., William F. Moore, III and Harvey L. Kincaid**
*SKBL-Tampa, SmithKline BioScience Laboratories Ltd, P.O. Box 22707, Tampa, Florida 33630, USA*

*An instrument evaluation of the Olympus AU5061 was conducted by a National Committee on Clinical Laboratory Standards (NCCLS, 771 East Lancaster Avenue, Villanova, Pennsylvania 19085, USA) protocol. Reagents having the same lot number were obtained from Data Medical Associates, Inc. (2016 East Randol Mill Road, Atlington, Texas 76011, USA). Formulations employed were those generally accepted as standard clinical chemistry methods. Control materials from Dade (American Hospital Supply Corporation, P.O. Box 520672, Miami, Florida 33152, USA) were analysed to determine within-run and day-to-day precision. Within-run precision (CV) was in the range of 0·31–4·6% for all methods and judged to be better than that of the SMAC II (Technicon Instruments, Tarrytown, New York 10591, USA). Linear ranges were equal or exceeded those available on the SMAC II. Of particular importance, the triglycerides method is linear up to 1000 mg/dl. Recovery studies demonstrated good recovery for all methods. Carry-over experiments did not demonstrate evidence of significant carry-over. Precision problems encountered with the bicarbonate, chloride and calcium methods were resolved by modifying the maintenance procedure. Throughput assessment demonstrated a maximum throughput of 280 specimens/h and an average throughput of 265 specimens/h. Significant savings in supplies over that required with the continuous flow analysers are achievable. Operator training is easily accomplished. A daily start-up routine requires approximately 1 h to complete and makes the instrument relatively easy to place it into test production.*

*Based upon the consistency of the analytical results obtained, its demonstrated throughput of 265 patient profiles/h, its ease of operation and the savings in operating expenses that are possible with the instrument, the AU5061 was judged to be an effective replacement for the SMAC II analyser.*

### Materials

*Quality-control materials*

Control material was obtained from Dade (American Hospital Supply Corporation, P.O. Box 520672, Miami, Florida 33152, USA). Monitrol I (Lot number XLS-37) and Monitrol II (Lot number XPS-128), which are unassayed lyophilized serum control materials were used for all precision measurements.

*Calibrators*

A 145/5·0 mmol/l aqueous standard containing lithium (3 mmol/l) as an internal standard was supplied by Olympus and used to calibrate the flame photometer. Set Point 1 (Lot number V6B258) and Set Point 2 (Lot number V6C261) from Technicon (Technicon Instruments Corporation, 511 Benedict Avenue, Tarrytown, New York 10591, USA) and the values supplied by Technicon were used to calibrate all other methods.

*New England Reagent Laboratory (NERL) Standards*

Weighed-in standards obtained from NERL (14 Almeida Avenue, East Providence, Rhode Island 02914, USA) were used to measure linearity for all chemistry procedures except the enzymes. For the latter, Multi-Enzyme Lin-Trol (PN M2266) was obtained from Sigma (Sigma Chemical Company, P.O. Box 14508, St Louis, Missouri 63171, USA) and diluted to obtain multiple points. For bilirubin, cholesterol, triglycerides, GGT and CK dilutions of a high patient serum were used to measure the linear response of these methods.

*Patient samples*

Patient samples which had been submitted to SKBL-Tampa for routine chemistry analysis were used for estimates of production capacity.

*Propane gas*

A special grade (99·5%) of propane gas was obtained from Bishop Welding Supply, Tampa, Florida and a 100 pound (net weight) tank was located outside the building to meet fire-code and safety requirements.

*Deionized water*

Laboratory grade deionized water was used throughout and was supplied by the in-house reverse osmosis/deionized water system.

*Chemistry reagents*

All reagents were supplied by Olympus and manufactured by DMA (Data Medical Associates, Inc., 2016 East Randol Mill Road, Arlington, Texas 76011, USA).

### Methods

Guidelines for clinical laboratory instrumentation evaluations followed those specified by National Committee on Clinical Laboratory Standards (NCCLS) [1–3].

*Chemistry methods*

All methods employ bichromatic measurements. The chemical basis for each method summarized below is taken from Olympus AU5000 application sheets [4]:

(1) *Na/K*: Flame photometry; lithium (3 mmol/l) is used as an internal standard and flame colour is measured at 589 nm (Na), 768 nm (K) and 671 nm (Li).

(2) *Glucose*: Hexokinase/G-6PDH coupled reaction; reduction of NAD is used to monitor the reaction bichromatically at 340/380 nm.

(3) *Chloride*: Mercuric thiocynate/ferric nitrate; colour is measured bichromatically at 520/600 nm.

(4) *BUN*: Urease/alpha-ketoglutarate; the oxidation of NADH to $NAD^+$ is measured bichromatically at 340/410 nm.

(5) *Creatinine*: Sodium picrate; colour is measured bichromatically at 520/600 nm.

(6) *Uric acid*: Uricase/peroxidase coupled reaction; colour of complex produced is measured bichromatically at 520/600 nm.

(7) *Calcium*: Cresolphthalein complexone and 8-hydroxyquinoline; colour is measured bichromatically at 570/600 nm.

(8) *Inorganic phosphorus*: Ammonium molybdate and sulfuric acid; unreduced phosphomolybdate complex is measured bichromatically at 340/380 nm.

(9) *Total protein*: Modified biuret reaction; colour of chromagen produced is measured bichromatically at 540/660 nm.

(10) *Albumin*: Bromcresol green at pH 4·2; colour is measured bichromatically at 600/750 nm.

(11) *Total bilirubin*: Formation of azobilirubin with 2,5 dichlorophenyldiazonium (2,5 DCPT); colour is measured bichromatically at 540/660 nm.

(12) *Alkaline phosphatase*: Substrate is p-nitrophenyl-phosphate in 2-amino-2-methyl-l-propanol; rate of reaction in the presence of magnesium is measured bichromatically at 410/520 nm.

(13) *Lactate dehydrogenase*: Substrate is lithium L-lactate; rate of reduction of NAD to NADH is measured at 340/410 nm.

(14) *AST* (*GOT*): Substrate is L-asparate/2-ketoglutarate, coupling with malate dehydrogenase (MDH); oxidation of NADH to NAD is measured at 340/410 nm.

(15) *ALT* (*GPT*): Substrate is L-alanine/2-ketoglutarate, coupling with lactate dehydrogenase (LD); oxidation of NADH to NAD is measured bichromatically at 340/410 nm.

(16) *Triglyceride*: Enzymatic coupling with lipase, glyerol kinase (GK), glycerol phosphate oxidase (GPO) and peroxidase (POD); 4 amminoantipyrine and 3-hydroxy-2,4,6-tribomobenzoic acid (TBHB) form a chromagen and colour is measured bichromatically at 540/600 nm.

(17) *Cholesterol*: Enzymatic coupling with cholesterol esterase, cholesterol oxidase and peroxidase; 4-aminoantipyrine and 3,4 dichlorophenol form a chromagen (quimoneimine) and colour is measured bichromatically at 520/750 nm.

(18) *Gamma Glutamyl Transferase* (*GGT*): Substrate is gamma-glutamyl-p-nitroanilide in glycylglycine; reaction rate is measured bichromatically at 410/520 nm.

(19) *Creatine kinase* (*CK*): Substrate is creatine phosphate, coupling with hexokinase and glucose-6-phosphodehydrogenase; rate of reaction is measured bichromatically at 340/520 nm. N-acetyl-L-cysteine (NAC) is included in the substrate formulation.

(20) *Iron*: Complex formation using 2,4,6 tripyridyl-s-triazine; colour is measured bichromatically at 600/750 nm.

### *Precision study*

The precision studies were conducted using two levels of control material. Within-run precision was determined from a set of 30 data points. Mean, standard deviation and coefficient of variation were calculated for each test. Day-to-day precision was determined for two 10-day sets and for the 20-day period overall.

### *Linearity study*

The linearity of each method was assessed using either weighed-in standards obtained from New England Reagent Laboratory (NERL), or, in the case of the enzymes, cholesterol, triglycerides and bilirubin, pooled patient sera. Multiple points throughout the dynamic range were measured.

### *Carry-over experiment*

In this experiment, 10 separate aliquots of the high and low controls were sampled to determine a random mean value for each control. This was compared to the carry-over mean value obtained for each. The latter was determined by alternating the sampling of the controls. A carry-over percentage was calculated as follows:

$$\text{Carry-over } (\%) = \frac{\text{Random mean} - \text{Carry-over mean}}{\text{Random mean}} \times 100$$

### *Correlation study*

Patient specimens ($N = 30$) were included in calculating correlation coefficients using the method of least squares. Values obtained on the SMAC II were compared to those obtained on the Olympus AU5061.

*Table 1. Olympus AU 5000 series of chemistry analysers.**

| Model | Number of channels | Samples/h | Tests/h |
|---|---|---|---|
| AU 5031 | 24 | 150 | 3600 |
| AU 5041 | 32 | 150 | 4800 |
| AU 5061 | 24 | 300 | 7200 |
| AU 5081** | 32 | 300 | 9600 |

* Manufacturer's specifications.
** Not available to the US market.

*Note:* Addition of the flame photometer for Na/K increases the number of channels (tests) by 2.

*Table 2. Within-run precision – control I.*

| Test | N | Mean | SD | Variance | CV.(%) | Range Min. | Range Max. |
|---|---|---|---|---|---|---|---|
| Glucose | 30 | 81.60 | 0.71 | 0.51 | 0.87 | 80 | 83 |
| Sodium | 30 | 150.13 | 0.62 | 0.38 | 0.41 | 148 | 152 |
| Potassium | 30 | 7.02 | 0.06 | 0.004 | 0.80 | 6.9 | 7.1 |
| Chloride | 30 | 107.07 | 0.73 | 0.53 | 0.68 | 106 | 108 |
| Bicarbonate | 30 | 22.77 | 0.92 | 0.85 | 4.04 | 21 | 24 |
| BUN | 30 | 15.93 | 0.25 | 0.06 | 1.57 | 15 | 16 |
| Creatinine | 30 | 1.14 | 0.05 | 0.002 | 4.24 | 1.1 | 1.2 |
| Uric Acid | 30 | 5.34 | 0.06 | 0.003 | 1.05 | 5.2 | 5.4 |
| Calcium | 30 | 9.30 | 0.15 | 0.022 | 1.58 | 9.0 | 9.7 |
| I. Phosphorus | 30 | 2.90 | 0 | 0 | 0 | 2.9 | 2.9 |
| T. Protein | 30 | 7.23 | 0.06 | 0.004 | 0.87 | 7.1 | 7.3 |
| Albumin | 30 | 4.29 | 0.02 | 0.001 | 0.58 | 4.2 | 4.3 |
| Bilirubin | 30 | 1.23 | 0.05 | 0.002 | 3.82 | 1.2 | 1.3 |
| Alk. Phos. | 30 | 49.80 | 0.54 | 0.29 | 1.09 | 49 | 51 |
| LDH | 30 | 102.67 | 1.37 | 1.89 | 1.34 | 101 | 105 |
| AST | 30 | 35.73 | 0.44 | 0.20 | 1.24 | 35 | 36 |
| ALT | 30 | 21.47 | 0.50 | 0.25 | 2.32 | 21 | 22 |
| Cholesterol | 30 | 239.83 | 1.19 | 1.41 | 0.49 | 237 | 241 |
| Triglyceride | 30 | 85.60 | 1.25 | 1.57 | 1.47 | 83 | 87 |
| Iron | 30 | 232.37 | 3.39 | 11.50 | 1.46 | 226 | 244 |
| GGT | 30 | 15.53 | 0.72 | 0.52 | 4.62 | 14 | 17 |
| CK | 30 | 157.13 | 2.72 | 7.38 | 1.73 | 150 | 162 |

*Table 3. Within-run precision – control II.*

| Test | N | Mean | SD | Variance | CV(%) | Range Min. | Range Max. |
|---|---|---|---|---|---|---|---|
| Glucose | 30 | 262.47 | 1.78 | 3.18 | 0.68 | 258 | 265 |
| Sodium | 30 | 117.07 | 0.36 | 0.13 | 0.31 | 116 | 118 |
| Potassium | 30 | 3.90 | 0.08 | 0.007 | 2.09 | 3.8 | 4.0 |
| Chloride | 30 | 85.67 | 0.60 | 0.36 | 0.70 | 85 | 87 |
| Bicarbonate | 30 | 15.23 | 0.56 | 0.31 | 3.67 | 14 | 16 |
| BUN | 30 | 51.53 | 0.62 | 0.38 | 1.20 | 50 | 52 |
| Creatinine | 30 | 6.13 | 0.06 | 0.004 | 1.04 | 6.0 | 6.2 |
| Uric Acid | 30 | 10.64 | 0.07 | 0.005 | 0.67 | 10.5 | 10.8 |
| Calcium | 30 | 13.01 | 0.15 | 0.024 | 1.18 | 12.7 | 13.4 |
| I. Phosphorus | 30 | 6.99 | 0.06 | 0.003 | 0.80 | 6.9 | 7.2 |
| T. Protein | 30 | 5.32 | 0.04 | 0.001 | 0.70 | 5.3 | 5.4 |
| Albumin | 30 | 3.33 | 0.05 | 0.002 | 1.38 | 3.3 | 3.4 |
| Bilirubin | 30 | 4.44 | 0.06 | 0.004 | 1.38 | 4.3 | 4.5 |
| Alk. Phos. | 30 | 234.30 | 2.79 | 7.81 | 1.19 | 230 | 244 |
| LDH | 30 | 342.37 | 4.09 | 16.77 | 1.20 | 336 | 348 |
| AST | 30 | 157.87 | 0.85 | 0.72 | 0.543 | 156 | 160 |
| ALT | 30 | 123.57 | 2.85 | 8.11 | 2.30 | 119 | 129 |
| Cholesterol | 30 | 121.60 | 0.76 | 0.57 | 0.62 | 120 | 123 |
| Triglyceride | 30 | 180.10 | 1.27 | 1.62 | 0.71 | 176 | 182 |
| Iron | 30 | 102.23 | 2.11 | 4.45 | 2.06 | 98 | 107 |
| GGT | 30 | 74.83 | 1.07 | 1.14 | 1.43 | 73 | 79 |
| CK | 30 | 568.00 | 7.24 | 52.40 | 1.27 | 551 | 584 |

*Throughput experiment*

The instrument was first primed with fresh reagents. Calibration samples and controls were placed on the instrument for initial set up and the instrument was re-calibrated every hour. Quality control samples were run every 300 patients. The instrument was operated continuously for a period of 5.3 hours during which 1410 patients were analysed. Four other throughput experiments consisted of from 500 to 600 specimens each.

### Results

*Within-run precision*

Data on within-run precision is summarized in tables 2 and 3. Precision for both levels of control material was

1–2% or less for most methods. Bicarbonate (4%) was a notable exception. At low concentrations, creatinine (4·2%) and bilirubin (3·8%) were somewhat higher than the other methods. The enzyme measurements showed remarkably good precision.

*Day-to-day precision*

Data on day-to-day precision is summarized in tables 4 and 5. Data was accumulated from 10 runs performed on 20 separate days. Group means were calculated from the daily means for each of two 10-day periods and overall for

*Table 4. Day-to-day precision – control I.*

| Test | N | Mean | SD | Variance | CV(%) | Range | |
|---|---|---|---|---|---|---|---|
| | | | | | | Min. | Max. |
| Glucose | 20 | 81·09 | 1·46 | 2·14 | 1·80 | 77 | 84 |
| Sodium | 20 | 149·45 | 1·35 | 1·83 | 0·90 | 144 | 154 |
| Potassium | 20 | 6·85 | 0·10 | 0·011 | 1·50 | 6·6 | 7·1 |
| Chloride | 20 | 106·09 | 1·44 | 2·08 | 1·36 | 102 | 110 |
| Bicarbonate | 20 | 22·29 | 1·26 | 1·60 | 5·67 | 19 | 25 |
| BUN | 20 | 15·88 | 0·52 | 0·28 | 3·31 | 14 | 18 |
| Creatinine | 20 | 1·12 | 0·05 | 0·003 | 4·72 | 1·0 | 1·3 |
| Uric Acid | 20 | 5·30 | 0·12 | 0·015 | 2·32 | 5·0 | 5·6 |
| Calcium | 20 | 9·43 | 0·24 | 0·056 | 2·51 | 8·8 | 10·1 |
| I. Phosphorus | 20 | 2·88 | 0·06 | 0·003 | 1·94 | 2·5 | 3·0 |
| T. Protein | 20 | 7·23 | 0·14 | 0·020 | 1·94 | 6·7 | 7·5 |
| Albumin | 20 | 4·33 | 0·05 | 0·002 | 1·13 | 4·2 | 4·5 |
| Bilirubin | 20 | 1·23 | 0·06 | 0·004 | 5·15 | 1·1 | 1·3 |
| Alk. Phos. | 20 | 53·08 | 2·48 | 6·13 | 4·67 | 49 | 60 |
| LDH | 20 | 99·24 | 4·09 | 16·71 | 4·12 | 92 | 108 |
| AST | 20 | 36·38 | 1·16 | 1·36 | 3·20 | 33 | 38 |
| ALT | 20 | 20·62 | 1·15 | 1·32 | 5·57 | 18 | 23 |
| Cholesterol | 20 | 237·13 | 3·61 | 13·03 | 1·52 | 225 | 247 |
| Triglyceride | 20 | 84·76 | 3·99 | 15·90 | 4·70 | 78 | 96 |
| Iron | 20 | 233·21 | 4·78 | 22·87 | 2·05 | 221 | 247 |
| GGT | 20 | 18·21 | 1·15 | 1·33 | 6·34 | 15 | 21 |
| CK | 20 | 166·58 | 10·07 | 101·40 | 6·05 | 150 | 193 |

*Table 5. Day-to-day precision – control II.*

| Test | N | Mean | SD | Variance | CV(%) | Range | |
|---|---|---|---|---|---|---|---|
| | | | | | | Min. | Max. |
| Glucose | 20 | 263·61 | 4·05 | 16·44 | 1·54 | 253 | 274 |
| Sodium | 20 | 116·68 | 1·01 | 1·03 | 0·87 | 113 | 119 |
| Potassium | 20 | 3·94 | 0·07 | 0·006 | 1·90 | 3·7 | 4·1 |
| Chloride | 20 | 85·02 | 1·28 | 1·64 | 1·51 | 81 | 87 |
| Bicarbonate | 20 | 16·33 | 1·16 | 1·35 | 7·12 | 13 | 18 |
| BUN | 20 | 51·50 | 0·92 | 0·84 | 1·78 | 49 | 53 |
| Creatinine | 20 | 6·15 | 0·12 | 0·014 | 1·94 | 5·8 | 6·4 |
| Uric Acid | 20 | 10·82 | 0·21 | 0·05 | 1·98 | 10·1 | 11·2 |
| Calcium | 20 | 13·17 | 0·37 | 0·14 | 2·84 | 12·2 | 14·0 |
| I. Phosphorus | 20 | 6·96 | 0·11 | 0·011 | 1·52 | 6·6 | 7·2 |
| T. Protein | 20 | 5·36 | 0·08 | 0·007 | 1·51 | 5·2 | 5·6 |
| Albumin | 20 | 3·39 | 0·04 | 0·002 | 1·24 | 3·3 | 3·5 |
| Bilirubin | 20 | 4·47 | 0·15 | 0·021 | 3·27 | 4·0 | 4·8 |
| Alk. Phos. | 20 | 243·00 | 6·04 | 36·49 | 2·49 | 229 | 258 |
| LDH | 20 | 319·53 | 13·63 | 185·86 | 4·27 | 293 | 348 |
| AST | 20 | 159·53 | 3·84 | 14·78 | 2·41 | 149 | 170 |
| ALT | 20 | 123·34 | 2·78 | 7·75 | 2·26 | 116 | 131 |
| Cholesterol | 20 | 121·92 | 1·42 | 2·02 | 1·17 | 117 | 127 |
| Triglyceride | 20 | 185·20 | 12·43 | 154·62 | 6·71 | 165 | 218 |
| Iron | 20 | 104·47 | 3·02 | 9·12 | 2·89 | 98 | 113 |
| GGT | 20 | 76·21 | 1·28 | 1·64 | 1·68 | 73 | 80 |
| CK | 20 | 568·14 | 15·44 | 238·31 | 2·72 | 522 | 593 |

the 20-day period. Precision on the bicarbonate procedure improved considerably by introducing a rinse solution containing hypochlorite. This was also found to be a necessary step with the chloride and calcium methods. Day-to-day precision for all methods, including enzymes, was less than 7%.

*Linearity*

Linearity of the methods was determined using weighed-in standards obtained from New England Reagent Laboratory (NERL), dilutions of patient sera, and serum based materials. These results are summarized in table 6. The stated dynamic range is equal to or wider than that for the SMAC, except for BUN and Iron. Comparison of linearity of methods for Olympus and the SMAC showed that the Olympus had a wider range for triglycerides, cholestrol, the enzymes, sodium and potassium. This comparison appears in table 7.

*Carry-over*

Results of the carry-over experiment are summarized in table 8. The amount of carry-over for sodium was between 1·26 and 2·54% and that for potassium was between 2·46 and 4·52%. Bicarbonate (3·77%) and creatinine (4·46%), calcium (2·43%) and bilirubin (5·33%) demonstrated a modest amount of carry-over. All the other methods had less than 1% carry-over and for some methods, i.e. glucose, ALT and BUN no evidence of carry-over was found.

*Correlation*

For most methods, patient values agreed with those of the SMAC II. Correlation coefficients above 0·900 were obtained, except for chloride, bicarbonate and calcium. For the latter, the range of values was too small to obtain a valid coefficient. The Olympus has the capability of adjusting its values to bring them into agreement with those obtained on another instrument. In this study, these factors were not used. Application of the factors can be made either before or after analysis is performed and final results can be made to mimic those of the SMAC II.

*Throughput*

The throughput achieved was 265 patient samples/hour and takes into account the time devoted to the set up, priming and calibration procedures required prior to patient sampling. The instrument is capable of achieving a maximum throughput of 280 samples/hour following

*Table 6. Linear range of methods.*

| Test | Source | Expected | Observed | Difference (%) |
|---|---|---|---|---|
| Glucose | NERL | 50 | 51 | 2·00 |
| | | 200 | 207 | 3·50 |
| | | 750 | 767 | 2·27 |
| Sodium | NERL | 100 | 103 | 3·00 |
| | | 130 | 132 | 1·54 |
| | | 160 | 160 | 0·00 |
| Potassium | NERL | 2·0 | 2·0 | 0·00 |
| | | 4·0 | 4·0 | 0·00 |
| | | 8·0 | 7·8 | −2·50 |
| Chloride | NERL | 70 | 72 | 2·86 |
| | | 100 | 102 | 2·00 |
| | | 130 | 126 | −3·08 |
| Bicarbonate | NERL | 5 | 5 | 0·00 |
| | | 25 | 24 | 4·00 |
| | | 40 | 39 | −2·50 |
| BUN | NERL | 10 | 9 | −10·00 |
| | | 50 | 50 | 0·00 |
| | | 70 | 68 | −2·86 |
| Creatinine | NERL | 1·0 | 1·0 | 0·00 |
| | | 7·0 | 7·0 | 0·00 |
| | | 15·0 | 15·0 | 0·00 |
| Uric acid | NERL | 4·0 | 4·2 | 5·00 |
| | | 8·0 | 8·3 | 3·75 |
| | | 12·0 | 12·1 | 0·83 |
| Calcium | NERL | 5·0 | 4·6 | −8·00 |
| | | 10·0 | 10·0 | 0·00 |
| | | 15·0 | 15·8 | 5·33 |
| I. phosphorus | NERL | 1·0 | 1·0 | 0·00 |
| | | 5·0 | 5·1 | 2·00 |
| | | 10·0 | 10·3 | 3·00 |
| T. protein | NERL | 2·0 | 2·1 | 5·00 |
| | | 6·0 | 6·4 | 6·67 |
| | | 10·0 | 10·7 | 7·00 |

these initial set-up procedures and assumes within-run calibration and quality control procedures are performed as required.

### Discussion

The instrument is designed for high sample and test throughput. The sample rate of the AU 5061 is fixed. This means that profiles of from 1 to 26 tests are performed at the same rate. The rate claimed by Olympus is 300/hour. The effective throughput taking into account start-up routines is actually 200/hour for the first hour and a maximum of 280/hour once the instrument is in full operation. Patient throughput was found to be 265 samples/hour. Other instrumental approaches consider test throughput rather than sample throughput. With the Olympus, a conservative throughput of 265 patient samples/hour and a maximum of 26 tests/sample gives a test throughput of 6890 tests/hour. This high throughput makes some special requirements on the steps devoted to specimen processing and data entry both prior to and following analysis. It is imperative that data entry is expedited and the flow of samples to the instrument optimized. Interface to a host computer facilitates the transfer of data.

The number of tests available on the AU 5061 is 26 and includes the two analytes on the flame (Na/K). This leaves 24 compared to 18 (20 – Na/K) on the SMAC II. The six available tests can be utilized effectively by offloading procedures done either manually or on smaller pieces of automated equipment. Additional savings in personnel and laboratory supplies can be achieved in this way. The software has a feature that provides objective photometric readings for assessing the degree of lipemia, icterus and hemolysis of the sample.

The instrument is fast. The throughput is two-and-a-half to three times that of the SMAC II analyser. The limiting factor is processing and essential data entry steps required for getting specimens onto the instrument. The linear range is wider in some cases which means fewer repeats. The 700 mg/dl upper limit for triglycerides, for instance, means that fewer repeats are required. Fewer repeats adds to the cost savings that can be realized.

*Table 6 continued.*

| Test | Source | Expected | Observed | Difference (%) |
|---|---|---|---|---|
| Albumin | NERL | 2·0 | 2·1 | 5·00 |
| | | 4·0 | 4·2 | 5·00 |
| | | 6·0 | 6·0 | 0·00 |
| Bilirubin | Pooled patient | 3·1 | 3·2 | 3·23 |
| | | 9·2 | 9·3 | 1·09 |
| | | 15·3 | 15·3 | 0·00 |
| Alk. phos. | Sigma | 391 | 409 | 4·60 |
| | | 783 | 805 | 2·81 |
| | | 1174 | 1174 | 0·00 |
| LDH | Sigma | 175 | 205 | 17·14 |
| | | 700 | 806 | 15·14 |
| | | 1750 | 1750 | 0·00 |
| AST | Sigma | 163 | 164 | 0·61 |
| | | 489 | 492 | 0·61 |
| | | 815 | 815 | 0·00 |
| ALT | Sigma | 221 | 225 | 1·81 |
| | | 662 | 668 | 0·91 |
| | | 1104 | 1104 | 0·00 |
| Cholesterol | Pooled patient | 105 | 108 | 2·86 |
| | | 314 | 311 | −0·96 |
| | | 524 | 524 | 0·00 |
| Triglyceride | Pooled patient | 205 | 219 | 6·83 |
| | | 411 | 412 | 0·24 |
| | | 821 | 821 | 0·00 |
| Iron | NERL | 50 | 50 | 0·00 |
| | | 150 | 144 | −4·00 |
| | | 300 | 285 | −5·00 |
| GGT | Pooled patient | 113 | 112 | −0·88 |
| | | 338 | 332 | −1·78 |
| | | 564 | 564 | 0·00 |
| CK | Pooled patient | 204 | 219 | 7·35 |
| | | 613 | 613 | 0·00 |
| | | 1022 | 1022 | 0·00 |

[1] NERL = New England Reagent Laboratory.

Temperature control for analytical measurements is achieved with a dry bath which surrounds the cuvette wheel. Coolant is circulated through an enclosed system and gives a constant fixed reaction temperature of 37 ± 0·2 °C. Variation of the reaction temperature to 25 °C or 30 °C for enzyme measurements is not possible. The glass reaction cuvette also serves as the measurement cuvette. Up to eight photometric measurements are taken at two wavelengths as the cuvettes advance. A series of fibre optics distribute the light from a single 100 W Halogen source lamp. A single interference filter wheel and eight photodiodes at each of the read stations completes the optical system. A second reagent addition occurs after the first reading for those methods such as the enzyme measurements which may require it. Both end-point and rate methods can be run and are selected by the individual test parameters (see table 9). Delayed readings can be specified for optimizing reaction rates for both end-point and kinetic methods. The test parameters include minimum and maximum absorbance values, reagent blanks, quality control, linear range and reference range. Sample blanks can be run but this requires devoting one of the available 24 tests to each blank method. Thus 12 blanks can be run on the AU 5061, together with 12 tests for a total of 24 available channels.

*Table 7. Stated dynamic ranges compared.*

| Test | Olympus | Units | SMAC II |
|---|---|---|---|
| Albumin | 6 | g/dl | 6 |
| Alk. phos. | 1500 | U/l | 750 |
| ALT | 500 | U/l | 500 |
| Amylase | 1000 | U/l | N/A |
| AST | 500 | U/l | 500 |
| Bicarbonate | 40 | mEq/l | 40 |
| T. bilirubin | 20 | mg/dl | 20 |
| D. bilirubin | 10 | mg/dl | N/A |
| BUN | 120 | mg/dl | 150 |
| Calcium | 16·0 | mg/dl | 15 |
| Chloride | 140 | mEq/l | 70–130 |
| Cholesterol | 500 | mg/dl | 500 |
| CK | 1500 | U/l | N/A |
| Creatinine | 20·0 | mg/dl | 20 |
| GGT | 1500 | U/l | 500 |
| Glucose | 500 | mg/dl | 500 |
| I. phosphorus | 15·0 | mg/dl | 10 |
| Iron | 1000 | ug/dl | 1250 |
| LD | 2000 | U/l | 600 |
| Magnesium | 4·5 | mEq/l | N/A |
| T. protein | 15·0 | g/dl | 10 |
| Triglyceride | 700 | mg/dl | 500 |
| Uric Acid | 20·0 | mg/dl | 15 |

The amount of reagent required for each determination is in the range of 250–500 μl which dramatically reduces the cost of reagents compared to that required to operate continuous-flow instruments. Table 9 summarizes method parameters for the various procedures. In addition, the cost of other consumables (pump tubing, coils etc.) is eliminated. Expenses for AU5061 consumables other than reagents include pump tubes to operate the flame, sample cups, and bar code labels. Other items would include reagent tubing, sample probes, and reaction cuvettes. During the brief 60-day evaluation period, none of the latter items needed replacement.

Approximately 80 $ft^2$ of floor space are required for installation and operation. Electrical requirements include 220 V (±10%), 50/60 Hz (±1 Hz), single phase grounded outlet. Other physical requirements include a floor drain, a source of deionized water capable of

*Table 8. An analysis of carry-over.*[1]

| Test | Random mean | Carry-over mean | Carry-over (percentage) | Random mean | Carry-over mean | Carry-over (percentage) |
|---|---|---|---|---|---|---|
| Glucose | 80·5 | 80·5 | 0·00 | 265·2 | 265·2 | 0·15 |
| Sodium | 151·2 | 149·3 | −1·26 | 118 | 121 | 2·54 |
| Potassium | 6·9 | 6·7 | −2·46 | 3·98 | 4·16 | 4·52 |
| Chloride | 106·1 | 105·8 | −0·28 | 84·5 | 85·0 | 0·59 |
| Bicarbonate | 23·9 | 24·8 | 3·77 | 18·5 | 18·5 | 0·00 |
| BUN | 15·8 | 15·8 | 0·00 | 50·8 | 50·6 | −0·39 |
| Creatinine | 1·12 | 1·07 | −4·46 | 6·03 | 6·14 | 1·82 |
| Uric acid | 5·27 | 5·29 | 0·38 | 10·43 | 10·45 | 0·192 |
| Calcium | 9·47 | 9·70 | 2·43 | 13·45 | 13·44 | −0·07 |
| Phosphorus | 2·91 | 2·89 | −0·69 | 6·95 | 6·98 | 0·43 |
| T. protein | 7·30 | 7·31 | 0·14 | 5·41 | 5·40 | −0·19 |
| Albumin | 4·30 | 4·32 | 0·47 | 3·40 | 3·39 | −0·29 |
| Bilirubin | 1·50 | 1·58 | 5·33 | 5·87 | 5·81 | −1·02 |
| Alk. phos. | 52·9 | 52·7 | −0·38 | 246·6 | 244·8 | −0·73 |
| LDH | 107·4 | 105·6 | −1·68 | 339·7 | 343·5 | 1·12 |
| AST | 35·5 | 35·7 | 0·56 | 157·2 | 155·7 | −0·95 |
| ALT | 20·0 | 20·0 | 0·00 | 121·7 | 120·4 | −1·07 |
| Cholesterol | 237·8 | 238·8 | 0·42 | 121·5 | 121·3 | −0·17 |
| Triglyceride | 82·1 | 82·6 | 0·61 | 175·3 | 174·0 | −0·74 |
| Iron | 234·1 | 232·5 | −0·68 | 104·5 | 105·5 | 0·96 |
| GGT | 17·1 | 16·9 | −1·17 | 74·5 | 74·9 | 0·54 |
| CK | 171·2 | 169·9 | −0·76 | 605·4 | 605·3 | −0·02 |

[1] Defined in the study as the amount of high (or low) control carried-over to a low (or high) control sample and expressed as a percentage.

delivering 60 l/h, and a supply of propane gas to operate the flame photometer.

Deionized water is required for blanking and washing the cuvettes. The water is de-gassed within the system to prevent air bubbles developing in any of the lines. The pumps required for this purpose create a certain amount of noise when they are in operation. However, they are shielded and the noise level was not considered a serious concern.

Test requisitions can be created on the system for up to 4000 patient specimens. This can be performed either before, during (STATs) or after the analyser is operational. Single tests or panels can be requisitioned for one sample (STATS) at a time or for groups of samples (batching). A work list is printed for the tests to be run. The instrument uses a sample number to indicate the order entry of the requisition and ties to it an accession or patient's identification number. Pre-defined panels are then requisitioned by a panel code number. Although these features are available, the high throughput of this instrument requires an interface program to a host computer to facilitate data transfer. Downloading of those key elements required to do the test (tests ordered, instrument ID, etc.) is necessary to optimize the work-flow. A simulation of this was tried with a personal

*Table 9. Method parameters.*

| Test | Sample volume (μl) | Reagent[1] volume (μl) | Method[2] | Wavelength[3] (nM) | Reference values |
|---|---|---|---|---|---|
| Albumin | 3 | 300 | EP | 600/750 | 3·5–5·0 g/dl |
| Alk. phos. | 4 | 250 | Rate | 410/520 | M: 30–125 U/l<br>F: 20–115 U/l |
| ALT | 10 | 200/50 | Rate | 340/410 | 5–30 U/l |
| Amylase | 6 | 250 | Rate | 410/520 | 25–125 U/l |
| AST | 10 | 200/50 | Rate | 340/410 | 0–34 U/l |
| D. bilirubin | 15 | 250/100 | EP | 540/660 | 0·0–0·35 mg/dl |
| T. bilirubin | 15 | 250 | EP | 540/660 | 0·1–1·5 mg/dl |
| Bicarbonate | 4 | 250/75 | EP | 380/410 | 22–33 mEq/l |
| Calcium | 3 | 125/125 | EP | 570/600 | 8·4–10·2 mg/dl |
| Chloride | 3 | 250 | EP | 520/600 | 98–106 mmol/l |
| Cholesterol | 3 | 300 | EP | 520/750 | 140–310 mg/dl |
| HDL-cholesterol | 12 | 300 | EP | 520/750 | M : 26–63 mg/dl<br>F : 33–75 mg/dl |
| CK | 5 | 250 | Rate | 340/520 | M : 38–174 U/l<br>F : 26–140 U/l |
| Creatinine | 15 | 250/125 | EP | 520/600 | M : 0·7–1·3 mg/dl<br>F : 0·6–1·2 mg/dl |

[1] Reagent 1/Reagent 2 when more than one reagent is used.
[2] EP = Endpoint; Rate = Kinetic method, usually with 3–6 readings.
[3] Primary/secondary wavelength for bichromatic analysis.

*Table 9 continued.*

| Test | Sample volume (μl) | Reagent[1] volume (μl) | Method[2] | Wavelength[3] (nM) | Reference values |
|---|---|---|---|---|---|
| GGT | 5 | 125/125 | Rate | 410/520 | M: 5–38 U/l<br>F: 5–29 U/l |
| Glucose | 3 | 300 | EP | 340/380 | 70–105 mg/dl |
| Iron | 15 | 250/150 | EP | 600/750 | M: 70–180 ug/dl<br>F: 60–180 ug/dl |
| I. phosphorus | 6 | 250/50 | EP | 340/380 | 2·5–4·5 mg/dl |
| LD | 5 | 250 | Rate | 340/410 | 100–225 U/l |
| Magnesium | 3 | 150/150 | EP | 520/750 | 1·3–2·1 mEq/l |
| T. protein | 4 | 250 | EP | 540/660 | 6·4–8·3 g/dl |
| Triglyceride | 3 | 250/50 | EP | 540/600 | M: 40–160 mg/dl<br>F: 35–135 mg/dl |
| BUN | 3 | 200/50 | EP | 340/410 | 7–18 mg/dl |
| Uric acid | 6 | 250/50 | EP | 520/600 | M: 3·5–7·2 mg/dl<br>F: 2·6–6·0 mg/dl |
| Sodium | 50 | N/A | ES | 589 | 136–146 mEq/l |
| Potassium | * | N/A | ES | 768 | 3·5–5·0 mEq/l |

[1] Reagent 1/Reagent 2 when more than one reagent is used.
[2] EP = Endpoint; Rate = Kinetic method, usually with 3–6 readings; ES = Emission Spectra.
[3] Primary/secondary wavelength for bichromatic analysis.
* Same sample as sodium.

computer and the bi-directional interface in the Olympus software was found to function according to specifications.

Correlation with SMAC II values was acceptable. The Olympus software has the capacity of adjusting its values to bring them into agreement with those obtained on the SMAC II. Adjustments can be made either before or after the tests are run and, for the latter, as a batch process. Reference values as supplied by Olympus are found in table 9 and differ from those standardized and employed by SKBL.

Calibration of the Olympus was conducted using SMAC reference sera. A calibrator supplied by Olympus was judged to be unacceptable. Definition of the number of calibrator and control samples is required. It is clearly different from that required for the SMAC II operation. Calibration is stable for up to 1 h for the flame, 2–3 h for bicarbonate, and 6–8 h for other chemistries. The stability of the instrument indicates that quality control checks may only be required every 300 patient samples. Considerable savings in quality-control material can thus be achieved with this instrument.

Reagents are stored in refrigerated compartments above the analytical components. The bottle size supplied was considered too small for high volume use. The container size should be increased four fold to accommodate 2 l for most chemistries. Checks on the existing reagent volumes are a part of the start-up protocol and this information is available through the CRT.

Maintenance is relatively simple to perform. The items requiring attention include the pump tubes on the flame. These should be replaced once a week. Weekly and monthly maintenance requires 30–40 min in each case. Every three months the conveyor belt and water tank need to be cleaned and this takes approximately 2 h to complete. The evaluation revealed that additional maintenance was required for the calcium, chloride and bicarbonate methods. This requires approximately 20 min/day and involves a cleaning of the reagent lines. The sample probe can become clogged with fibrin clots and must be visually inspected during operation. The instrument performs a forced flush with deionized water between samples which helps to minimize this occurrence. Additionally, time must be devoted to preparing the samples which includes filtering all samples. Replacement of the sample probe is not difficult. The sample probe is a vital link. Since each module contains its own probe, a malfunction in one probe effects only the four tests on that module.

The computer system offers only rudimentary quality-control features. This function is better handled using more sophisticated programs available on a laboratory host computer. The options available include calculations for standard deviation, mean, and coefficient of variation. It will not allow elimination of outliers, and editing of QC data is not easily performed. It does provide QC charts for each test and each control individually, but decision-making trees and summary reports are not included.

The instrument for evaluation was not interfaced to the laboratory computer. Testing of the bi-directional interface using a PC indicated that it did perform as specified by Olympus. The conclusion reached was that an interfaced program could be written and interfaced to the SKBL host computer system.

The bar-code reading device was not functional with the labels supplied. A source of bar-code labels was obtained and the labels were placed on the rack instead of the tubes. This allows for the re-use of the label and was considered a better approach than placing it on the tube. The instrument requires entry of specimen identification before analysis is performed. The Olympus defaults to not doing any tests but only for that particular sample. As a result, the instrument does not stop completely if a patient identification has not been entered. It will continue to analyse the other specimens that have been entered.

Olympus has indicated that the current software programs are to be enhanced with special packages for quality control, result verification using a multi-variate alogorithm and management reporting. The calculated parameters available at this time are A (B), (A-B)/A, A/B and A/(B-A). It does not permit, for instance, the calculation of an ionized calcium. The bar code reading device worked with the code-a-bar (European) format. It uses an LED device and has the advantage that it does not have any moving parts. Olympus has indicated further that a dial-up capability for diagnostics will be offered.

Training is easily accomplished. Operator proficiency can be achieved in a shorter period than that required to operate a SMAC II. The instrument is menu driven by the CRT and is easy to follow. Help screens are not a part of the software program, however. Trouble shooting is easier to perform using a much improved and updated operator's manual.

### Conclusion

The instrument did not have any serious problems during the 60-day evaluation period that caused it to be down. Overall, the Olympus AU5061 was judged to be an effective replacement for the SMAC II instrument providing advantages in increased throughput with significant reductions in operating expenses.

### References

1. *User Evaluation of Precision Performance of Clinical Chemistry Devices,* Vol. 4, No. 8 (Order Code EP5-T, NCCLS, Villanova, PA 19085, USA).
2. *User Comparison of Quantitative Clinical Laboratory Methods Using Patient Samples,* Vol. 6, No. 1 (Order Code EP9-P, NCCLS, Villanova, PA 19085, USA).
3. *Minimum Acceptability Performance Evaluation of Clinical Chemistry Methods,* Vol. 6, No. 3 (Order Code EP10-P, NCCLS, Villanova, PA 19085, USA).
4. *Description of Methodologies for Olympus AU5000 Application* (Olympus Corporation, Lake Success, New York 11042, USA).

